# All-stage precisional glioma targeted therapy enabled by a well-designed D-peptide

**DOI:** 10.7150/thno.41382

**Published:** 2020-03-04

**Authors:** Danni Ran, Jianfen Zhou, Zhilan Chai, Jinyang Li, Cao Xie, Jiani Mao, Linwei Lu, Yanyu Zhang, Sunyi Wu, Changyou Zhan, Weiyue Lu

**Affiliations:** 1 Department of Pharmaceutics, School of Pharmacy, Fudan University, and Key Laboratory of Smart Drug Delivery (Fudan University), Ministry of Education and PLA, Shanghai 201203, & State Key Laboratory of Medical Neurobiology and MOE Frontiers Center for Brain Science, Institutes of Brain Science, Fudan University, Shanghai 200032, China.; 2 Department of Integrative Medicine, Huashan Hospital, and Institutes of Integrative Medicine of Fudan University, Shanghai 200041, China.; 3 Department of Pharmacology, School of Basic Medical Sciences, Fudan University, Shanghai 200032, & State Key Laboratory of Molecular Engineering of Polymers, Fudan University, Shanghai 200433, China.; 4 Minhang Branch, Zhongshan Hospital and Institute of Fudan-Minghang Academic Health System, Minghang Hospital, Fudan University, Shanghai 201199, China.

**Keywords:** ^D^WVAP, all-stage targeting, drug delivery, multiple biological barriers, glioma, glioma stem cells

## Abstract

Uncontrollable cell proliferation and irreversible neurological damage make glioma one of the most deadly diseases in clinic. Besides the multiple biological barriers, glioma stem cells (GSCs) that are responsible for the maintenance and recurrence of tumor tissues also hinder the therapeutic efficacy of chemotherapy. Therefore, all-stage precisional glioma targeted therapy regimens that could efficiently deliver drugs to glioma cells and GSCs after overcoming multiple barriers have received increasing scrutiny.

**Methods**: A polymeric micelle-based drug delivery system was developed by modifying a “Y-shaped” well-designed ligand of both GRP78 protein and quorum sensing receptor to achieve all-stage precisional glioma targeting, then we evaluated the targeting ability and barrier penetration ability both *in vitro* and *in vivo*. In order to achieve all-stage precisional therapy, we need kill both GSCs and glioma related cells. Parthenolide (PTL) has been investigated for its selective toxicity to glioma stem cells while Paclitaxel (PTX) and Temozolomide (TMZ) are widely used in experimental and clinical therapy of glioma respectively. So the *in vivo* anti-glioma effect of combination therapy was evaluated by Kaplan-Meier survival analysis and immunohistochemical (IHC) examination of tumor tissues.

**Results**: The “Y-shaped” well-designed peptide, termed ^D^WVAP, exhibited excellent glioma (and GSCs) homing and barrier penetration ability. When modified on micelle surface, ^D^WVAP peptide significantly enhanced accumulation of micelles in brain and glioma. In addition, ^D^WVAP micelles showed no immunogenicity and cytotoxicity, which could guarantee their safety when used *in vivo*. Treatment of glioma-bearing mice with PTL loaded ^D^WVAP modified PEG-PLA micelles plus PTX loaded ^D^WVAP modified PEG-PLA micelles or PTL loaded ^D^WVAP modified PEG-PLA micelles plus TMZ showed improved anti-tumor efficacy in comparison to PTL and PTX loaded unmodified micelles or PTL loaded unmodified micelles plus TMZ.

**Conclusion**: Combination of all-stage targeting strategy and concomitant use of chemotherapeutics and stem cell inhibitors could achieve precise targeted therapy for glioma

## Introduction

**Figure 1:**
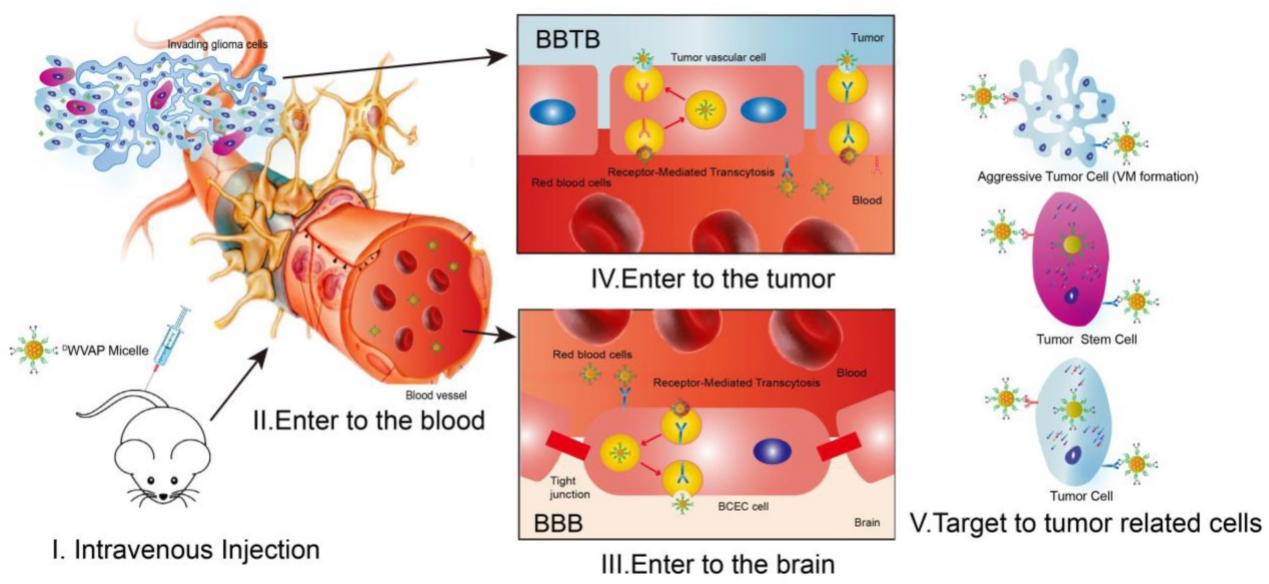
Schematic illustration of the main obstacles to anti-glioma drug delivery and the concept of the all-stage precisional glioma targeting process. Peptide ligand functionalized micelles are designed to transverse the blood-brain barrier (BBB), blood-brain tumor barrier (BBTB) and destruct vasculogenic mimicry (VM), then target glioma cells and glioma stem cells.

Glioblastoma multiforme (GBM) is one of the most common and devastating diseases in the central nervous system; and about 14,000 patients are annually diagnosed with GBM in the USA 1. GBM accounts for 12% to 15% of all intracranial tumors and 50% to 60% of astrocytic tumors 2,3. Despite the fact that GBM rarely spreads outside the brain, this infiltrative characteristic makes it difficult to be cured by surgical resection 4. Chemotherapy has become indispensable in clinic for GBM patients after surgery. However, multiple biological barriers severely hamper the delivery of chemotherapeutics to glioma region after systemic administration 5,6 (as depicted in Figure 1). The blood-brain barrier (BBB) is a physical and biochemical barrier that plays pivotal roles in maintaining brain homeostasis; while it also presents an obstacle to deliver most drugs into the brain, severely restricting brain transport of anti-glioma therapeutics at the early stages and to the center of glioma at the advanced stages 7. Even though angiogenesis is very common in glioma recurrence, the special microenvironment in the brain leads to relatively narrower fenestrae in glioma neovasculature than that of peripheral tumors, forming the so-called blood-brain tumor barrier (BBTB), which impedes the penetration of drug carriers into glioma tissues 8,9. Moreover, vasculogenic mimicry (VM) formed by invasive glioma cells instead of endothelial cells and angiogenesis also relates to the poor prognosis and recurrence of glioma 10. The last but not the least intractable obstacle is inaccessibility of drugs to the glioma stem cells (GSCs), which are responsible for the tumorigenesis and recurrence of GBM 11-14. Thus, it is a clinical desire to develop an all-stage precisional glioma targeted therapeutic regimen that can make drug penetrate the BBB, BBTB and destruct VM, then target the glioma cells and GSCs to boost the anti-glioma effect of chemotherapeutics to the most extent.

**Figure 2:**
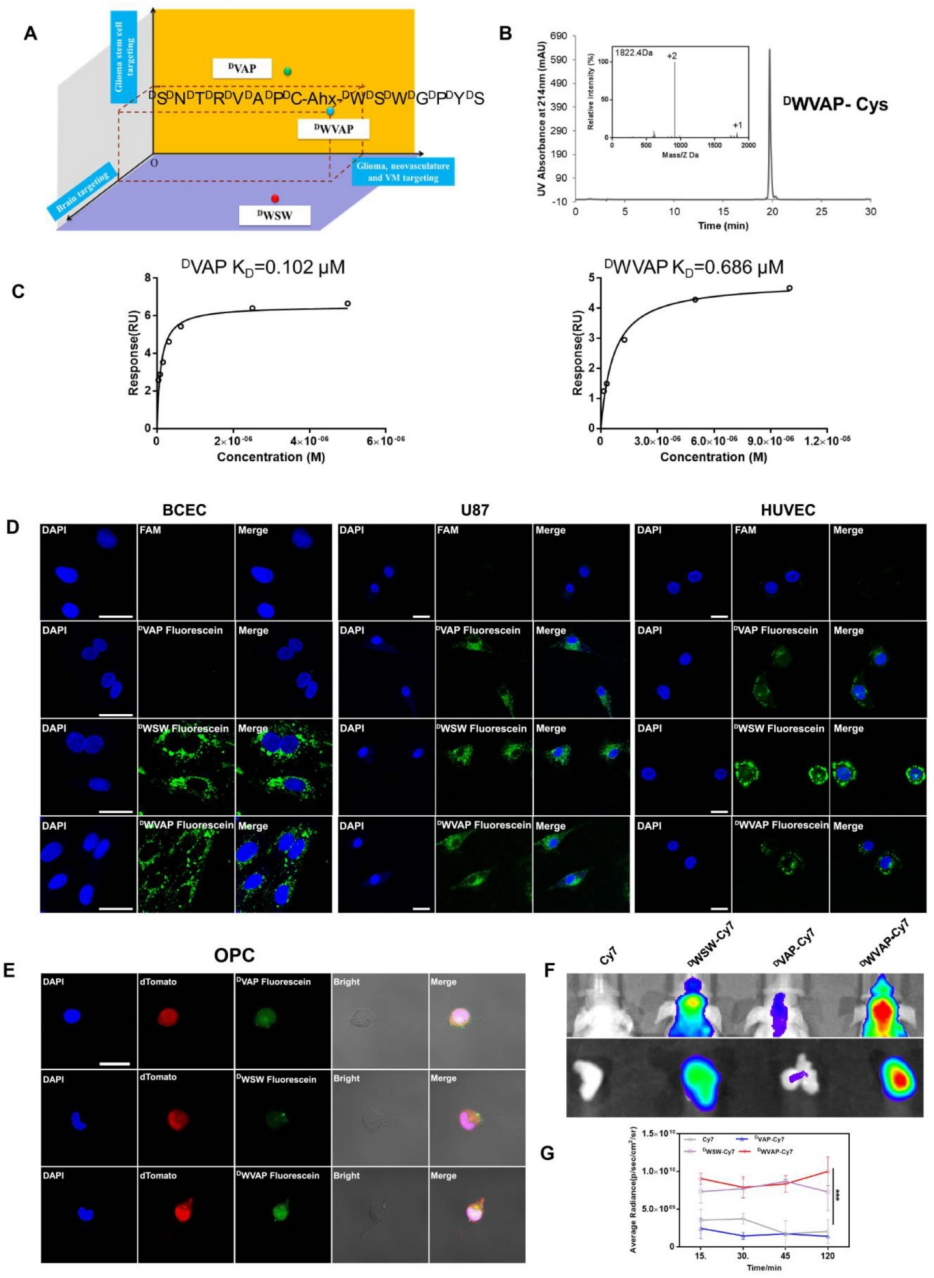
Designation and characterization of ^D^WVAP peptide. (A) Schematic illustration of the design of ^D^WVAP peptide. (B) The HPLC and MS spectrum of ^D^WVAP-Cys. (C) Interaction of GRP78 protein with ^D^VAP and ^D^WVAP. Data were collected with Biacore control software version 2.0 and analyzed by Biacore T200 evaluation software. All peptides were prepared in HBS-N buffer in a 2-fold serial dilution and injected onto the GRP78 protein-immobilized CM5 sensor chip at a flow rate of 30 µL/min. (D) Targeting ability of various peptides in BCEC cells, U87 cells and HUVEC cells evaluated by confocal microscope, scale bar = 10 µm. (E) Targeting ability of various peptides in OPC cells observed by confocal microscopy, scale bar is 10 µm. (F) *In vivo* imaging of biodistribution of Cy7-labeled peptides in brains and *ex vivo* imaging of dissected tumor 2 h after injection. (G) Normalized fluorescence intensity of brains in each group at 15, 30, 45 and 120 min. Mean ± SD, n = 3. *** p < 0.001.

The therapeutic regimen can be divided into two parts. First, all-stage precisional targeting, a variety of peptide ligands have been identified in the previous reports to penetrate BBB, BBTB and/or target to VM by receptor-mediated transcytosis 15-19. Among those receptors, GRP78 is a cancer cell-surface marker overexpressed on glioma cells, VM, neovasculature and GSCs but not on normal cells 19, 20. ^D^VAP (^D^S^D^N^D^T^D^R^D^V^D^A^D^P) is a stable peptide possessing high binding affinity to GRP78 protein 19. It is a versatile peptide ligand for multifunctional glioma-targeted drug delivery (e.g. penetrating BBTB and VM, then targeting glioma cells and GSCs), except that BBB still presents a rigid obstacle to the delivery of ^D^VAP functionalized nanocarriers to the brain. It has been reported that quorum-sensing peptide ^D^WSW (^D^W^D^S^D^W^D^G^D^P^D^Y^D^S) can selectively transverse BBB. In our previous study, we found that connecting two peptides through aminocaproic acid to form a "Y" shape structure could increase the modification density of the targeting peptide in one PEG-PLA molecule and retain the functions of both peptide 21. Thus, we hypothesize that a “Y-shaped” ^D^VAP-derived ligand termed ^D^WVAP (Figure 2A) that can also penetrate the BBB would be an ideal molecule for achieving all-stage precisional glioma targeting. In this work we designed a multifunctional D peptide in aim to achieve all-stage precisional glioma targeting as illustrated in Figure 1. We evaluated its targeting ability to glioma cells, glioma stem cells and glioma related vascular cells. Then, we solved the all-stage precisional therapy through combination therapy. Paclitaxel has been reported to be encapsulated into PEG-PLA micelles for glioma therapy 22 and temozolomide (TMZ) has been applied as the first-line treatment of glioma 23. We choose parthenolide because of its unique GSCs toxicity and it is a potent inhibitor of NF-κB pathway 24. The anti-glioma effects of parthenolide-loaded micelles (^D^WVAP-M/PTL) and paclitaxel-loaded micelles (^D^WVAP-M/PTX) were evaluated in an intracranial glioma-bearing nude mice model. Then, ^D^WVAP-M/PTL was used together with ^D^WVAP-M/PTX or TMZ respectively and achieved promising therapeutic efficacy. Meanwhile, the underlying mechanism of the combination therapy was also validated.

## Results and Discussion

### Characterization of the well-designed ^D^WVAP peptide

The brain targeted D-peptide ligand for quorum sensing receptor, ^D^WSW (^D^W^D^S^D^W^D^G^D^P^D^Y^D^S) 25, was conjugated with ^D^VAP peptide (^D^S^D^N^D^T^D^R^D^V^D^A^D^P) 19 to generate the “Y-shaped” novel peptide ligand possessing the potential to achieve all-stage precisional glioma targeting, termed ^D^WVAP, via an aminocaproic acid linker (Figure 2A). Mass spectrum and HPLC chromatograph of ^D^WVAP and its derivatives were displayed in Figure 2B. To investigate whether ^D^WVAP is capable of interacting with GRP78 protein, surface plasmon resonance assay was conducted to quantify the binding affinity. Recombinant human GRP78 protein was coupled to the CM5 sensor chip according to the manufacture's introduction. Both ^D^VAP and ^D^WVAP could bind to GRP78 protein in a dose-dependent manner (Figure 2C). ^D^WVAP and ^D^VAP registered K_D_ values of 686 nM and 102 nM, indicating that conjugation of^ D^WSW and ^D^VAP via an aminocaproic acid linker only slightly decreased the binding affinity of ^D^VAP with GRP78 protein.

### Targeting ability of ^D^WVAP peptide

The targeting ability of ^D^WVAP was evaluated in primary rat brain capillary endothelial cells (BCECs), HUVECs and U87 cells. As shown in Figure 2D, both ^D^WVAP and ^D^WSW could be efficiently taken up by BCECs. More than 90% of U87 cells and 80% of HUVECs were fluorescence-positive after incubation with ^D^VAP, ^D^WSW and ^D^WVAP (Figure S1), suggesting that ^D^WVAP was endowed with brain targeting ability while maintaining tumor homing ability. OPC cells were previously proven as a cell line of glioma cell-of-origin with under-appreciated proliferative and plastic potentials 19. OPC cells were used here to evaluate the GSCs targeting effect of the developed peptides. As shown in Figure 2E, both ^D^WVAP and ^D^VAP could be effectively taken up by OPC cells. These results verified that only^ D^WVAP could simultaneously target BCECs, HUVECs, glioma cells and GSCs. *In vivo*, near-infrared dye Cy7 was conjugated with peptides and the conjugates were intravenously injected via the tail vein to test the targeting effect of ^D^WVAP. It was evident that ^D^WVAP-Cy7 displayed significantly higher distribution in U87 glioma region in comparison to ^D^VAP-Cy7 (Figure 2F-G).

### Characterization of ^D^WVAP-modified micelles

Peptide was conjugated with Mal-PEG_3000_-PLA_2000_ through the thiol-maleimide addition reaction. In the NMR spectrum of Mal-PEG_3000_-PLA_2000_, the maleimide group exhibited a peak at 7.0 ppm, while the peak completely disappeared in that of ^D^WVAP-PEG_3000_-PLA_2000_, indicating successful reaction between the maleimide group and the thiol group of peptide (Figure S8). The particle size of micelles was around 30 nm. Meanwhile, the size distribution was confirmed by transmission electron microscope (Figure S3). The particle size, encapsulation efficiency and drug loading capacity were nearly similar among those different micelle formulations containing the same drug (Table S1), indicating that the modification of ^D^WVAP peptide may not affect the physical characteristics of PEG-PLA micelles.

### Barrier penetration ability of ^D^WVAP peptide modified micelles

**Figure 3:**
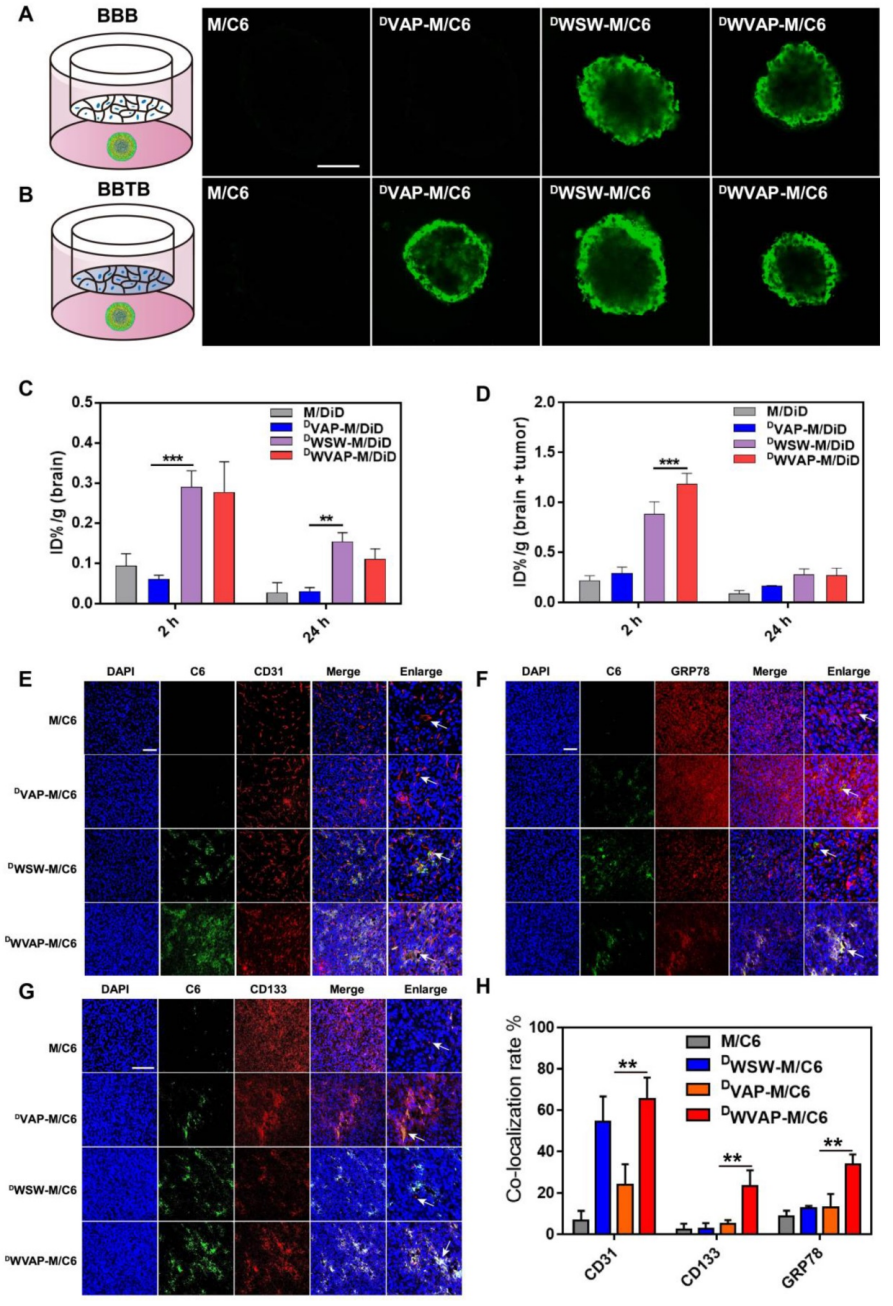
Evaluation of the BBB and BBTB penetrating capacity *in vitro* and the brain and glioma targeting ability* in vivo* in mice. (A) Schematic illustration of the BBB using BCEC monolayer and U87 tumor spheroid co-culture model. Uptake of Coumarin-6-loaded plain micelle, ^D^VAP Micelle, ^D^WSW Micelle and ^D^WVAP Micelle was imaged by confocal microscopy. (B) Schematic illustration of the BBTB using HUVEC monolayer and U87 tumor spheroid co-culture model. Uptake of Coumarin-6-loaded plain micelle, ^D^VAP Micelle, ^D^WSW Micelle and ^D^WVAP Micelle was imaged by confocal microscopy. (Bar = 250 µm). (C) *In vivo* distribution of DiD labeled plain micelles and peptides modified micelles in the excised brain of normal mice 2 h, and 24 h after injection. (D) *In vivo* distribution of DiD labeled plain micelles and peptides modified micelles in the excised brain of intracranial tumor bearing mice 2 and 24 h after injection (mean ± SD). Distribution of various micelle formulations in the brains of nude mice bearing U87 glioma determined by a confocal laser microscope. Red: anti-CD31 antibody (E), anti-GRP78 antibody (F), anti-CD133 antibody (G). Blue: DAPI. Green: Coumarin-6 labeled micelles. (H) Statistical analysis of different micelle formulations co-localized with two different markers CD31, GRP78 and CD133. The co-localization rate was calculated by Image-Pro Plus software. Scale bar is 100 µm. Mean ± SD, n = 3. ** p < 0.05, *** P < 0.001.

Polymeric micelles are a class of versatile nanocarriers for the delivery of a variety of chemotherapeutics. We prepared ^D^WVAP peptide modified PEG-PLA polymeric micelles to assess the targeting ability. *In vitro* BBB model was constructed using primary BCECs to evaluate the trans-BBB efficiency of the micelles; while the BBTB model was constructed as previously reported 17 to assess the trans-BBTB efficiency of the micelles. As shown in Figure S2, both ^D^WVAP and ^D^WSW modification significantly boosted the transcytosis efficiency of micelles across the BBB and all peptides decoration enhanced transcytosis efficiency across the BBTB. In order to better mimic the situation of glioma both at the early and advanced stages, BBB (BBTB)/U87 tumor spheroids co-culture models were established to assess the targeting ability of ^D^WVAP modified micelles *in vitro*. As shown in Figure 3A, the fluorescence intensities of tumor spheroid treated with ^D^WVAP-M/C6 and ^D^WSW-M/C6 were higher than that of tumor spheroid treated with^ D^VAP-M/C6 in the BBB/U87 tumor spheroids model. However, in Figure 3B since all peptides decoration could enhance micelles transcytosis efficiency across the BBTB, the fluorescent intensity in all three peptides modified micelles did not exhibit obvious difference. The *in vivo* brain and glioma targeting ability of ^D^WVAP peptide modified micelles were assessed in normal mice and intracranial U87 glioma-bearing nude mice 14 days after tumor implantation respectively. It was evident that both in normal mice and glioma-bearing mice ^D^WVAP modification induced high brain distribution of micelles (Figure 3C, D).

### *In vivo* targeting ability of ^D^WVAP peptide modified micelles

To assess the biodistribution and GSCs targeting efficacy of ^D^WVAP modified micelles *in vivo*, immunofluorescence analysis of the frozen brain sections was conducted. Coumarin-6 loaded ^D^WVAP modified micelles demonstrated higher accumulation in the glioma region than did M/C6 and ^D^VAP-M/C6 (Figure 3E, F and G). ^D^WVAP-M/C6 were co-localized with tumor blood vessel marker CD31 (Figure 3E), indicating that ^D^WVAP modified micelles could target to tumor neovasculature. As expected, ^D^WVAP modified micelles were mostly co-localized with GRP78 protein (Figure 3F) overexpressed in tumor cells, tumor stem cells, VM and angiogenesis. CD133 was used as a classic marker for GSCs. As shown in Figure 3G and H, co-localization of C6 with CD133 positive glioma cells (GSCs) could be observed in the glioma region of ^D^WVAP-M/C6 treated mice, indicating that ^D^WVAP peptide could efficiently achieve GSCs targeting after crossing multiple biological barriers.

### Immune response characterization of ^D^WVAP peptide modified micelles

**Figure 4:**
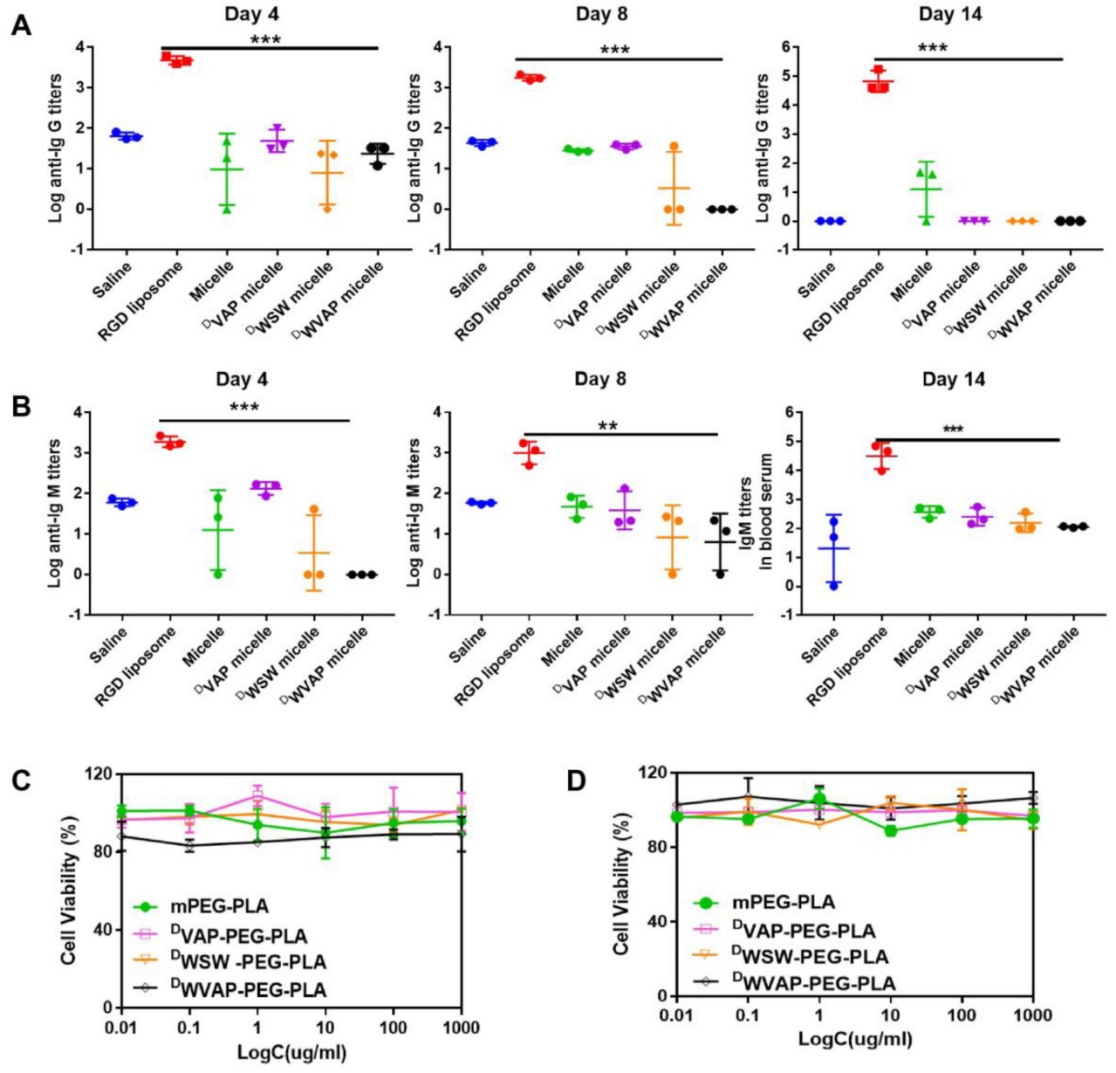
Safety evaluation of ^D^WVAP peptide modified micelles. (A) Serum IgG, (B) IgM production in ICR mice at day 4, day 8 and day 14 after injection of various formulations at day 0, day 3 and day 6 (Mean ± SD, n=3), ** p < 0.01, *** p < 0.001. Effect of various functional micelle materials on the proliferation of U87 cells (C) and HUVEC cells (D) by MTT assay.

The ability of the ^D^WVAP peptide modified micelles to elicit potent humoral immunity was studied. Compared to saline (the negative control group), the c(RGDyK)-liposomes elicited both significant Ig G and Ig M titers in mice serum, which proved the validity of the method. As shown in Figure 4A and B, in the ^D^WVAP peptide modified micelles group, the titers of both Ig G and Ig M were of no difference when compared with saline group, which indicated that it would not elicit immune response when used *in vivo*.

### *In vitro* anti-glioma efficacy of drug loaded ^D^WVAP peptide modified micelles

**Figure 5:**
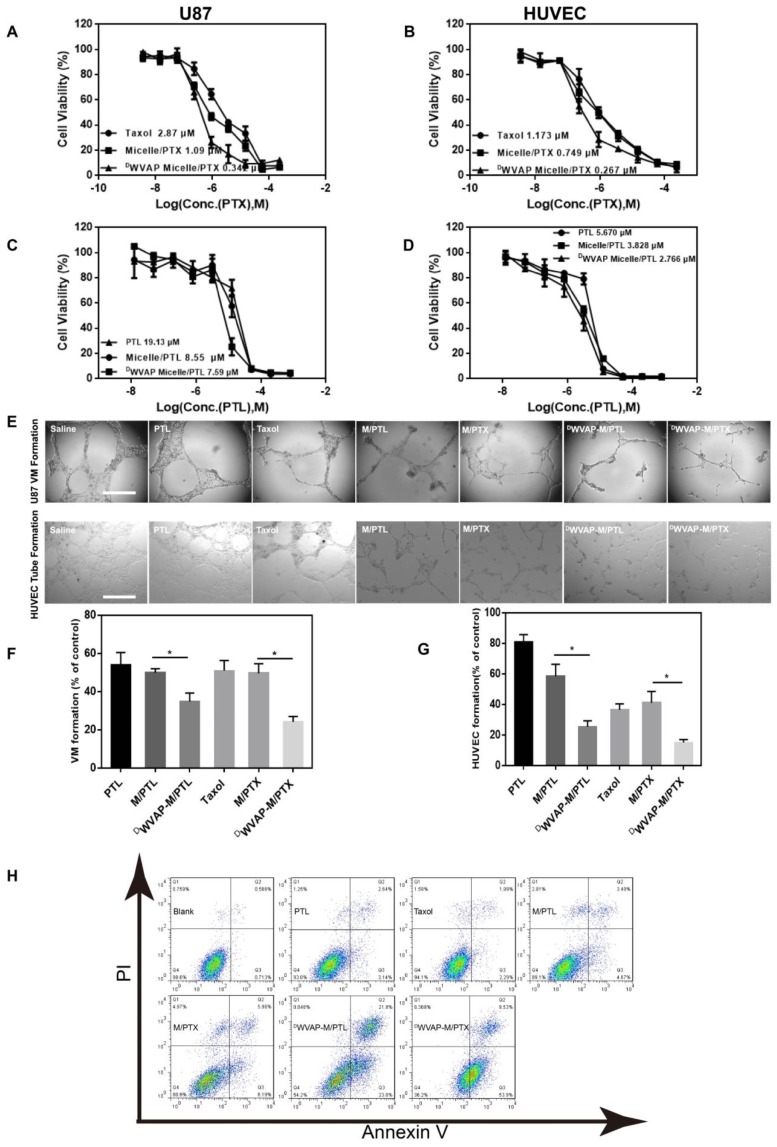
*In vitro* anti-glioma efficacy of drug loaded ^D^WVAP peptide modified micelles. Effect of various formulations on the proliferation of U87 cells (A and C) and HUVEC cells (B and D) by MTT assay. Tube formation assay was performed to assess the effect of various formulations on U87 VM formation and HUVEC tube formation (E). The quantitative data of the U87 VM formation (F) and HUVEC tube formation (G) treated by various formulations. Cells treated with drug-free DMEM served as the control. Data were presented as the percentages of the control group, which was set at 100%. *p < 0.05. (H) Apoptosis analysis of U87 cells after different treatments.

To verify our concept of all-stage precisional glioma targeted therapy regimen, we use U87 cells, HUVEC cells as *in vitro* model to evaluate the therapeutic efficacy. First, we confirmed that ^D^WVAP-PEG-PLA do no harm to U87 and HUVEC cells (Figure 4C and D). Paclitaxel and parthenolide were chosen to kill tumor related cells and glioma stem cells respectively. We then tested the anti-glioma efficacy of PTX loaded ^D^WVAP micelles. *In vitro*, ^D^WVAP Micelle/PTX displayed anti-proliferative effect on U87 cells and HUVEC cells (Figure 5A and B), it could also prevent them forming vessel like structures (Figure 5E and F). However, paclitaxel is considered ineffective in inhibiting the proliferation of GSCs. Parthenolide (PTL) is a small molecule selectively killing cancer stem cells while sparing normal counterparts 26,27; however its anti-cancer stem cell efficacy is usually hampered by high hydrophobicity and low stability 28. For anti-glioma therapy, the clinical use of PTL is further hindered by the multiple biological barriers. Here, the anti-glioma efficacy of PTL itself and PTL loaded micelles was evaluated. ^D^WVAP Micelle/PTL could induce more U87 apoptosis than did Micelle/PTL (Figure 5H) and inhibit the proliferation of U87 cells as well as HUVEC cells, and destroy the vessel-like structure formation (Figure 5C, D and G).

### *In vivo* anti-glioma efficacy and combination therapy

The intracranial glioma bearing mice were used to evaluate the anti-glioma efficacy of various therapeutic regimen. We first verified that micelles modified with the ^D^WVAP peptide exhibited an improved anti-glioma effect compared to that of ^D^WSW micelles and ^D^VAP micelles (Figure S4). As shown in Figure S5A, treatment of ^D^WVAP Micelle/PTX (median survival time = 29.5 days) could significantly improve the anti-glioma effect of paclitaxel in comparison to the free drug (median survival time = 20 days) and unmodified micelles (median survival time = 21 days). As shown in Figure S5B, in the absence of ^D^WVAP peptide, treatments with free or micelle-formulated PTL at a dose of 5 mg per kg body weight (at 6, 8, 10, 12 and 14 days post-tumor implantation) did little in improving mouse survival, registering the median survival of both 32 days versus 29.5 days of the saline treated group. Treatment of ^D^WVAP Micelle/PTL (37 days, p=0.0305) significantly lengthened the survival of mice with 2-fold prolonged median survival time than that of micelles without ^D^WVAP modification. All these results indicated that ^D^WVAP modification could prolong the average survival time of nude mice model.

**Figure 6:**
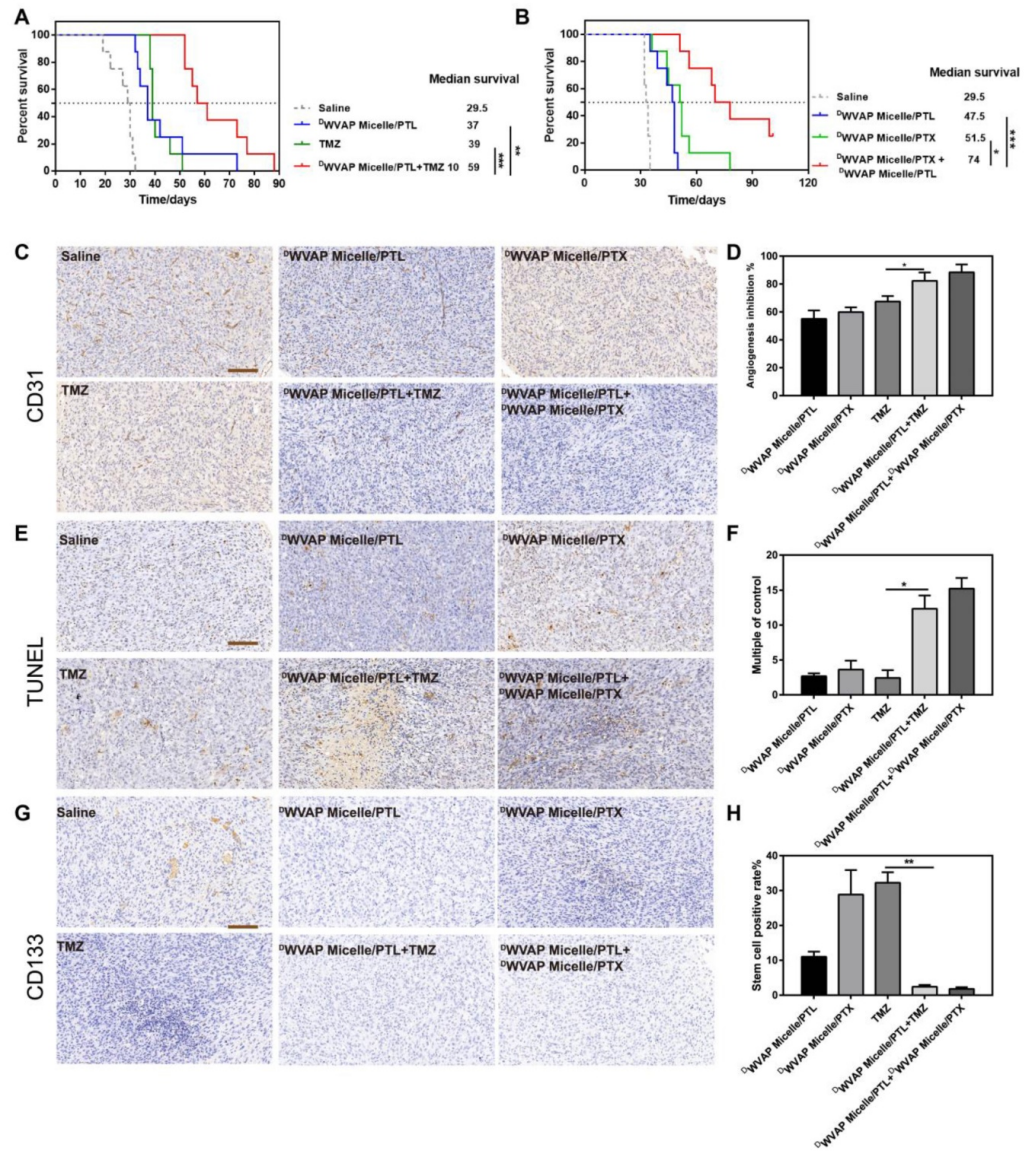
Kaplan-Meier survival curves of intracranial glioblastoma-implanted nude mice treated with different regimens. (A) Mice (n = 8) were treated with TMZ (10 mg/kg) alone or together with ^D^WVAP-M/PTL (5 mg/kg with respect to PTL). TMZ was administered through oral gavage while PTL was given by caudal vein on the 6th, 8th, 10th, 12th and 14th day after implantation. (B) Mice were treated with ^D^WVAP-M/PTX (6 mg/kg with respect to PTX) alone or together with ^D^WVAP-M/PTL (5 mg/kg with respect to PTL). All formulations were administered through caudal vein for all mice on the day on the 6th, 9th, 12th, 15th and 18th day after implantation. Mean ± SD, n = 8. ** p < 0.01, *** p < 0.001. Immunohistochemical analysis of the tumor tissues of the mice bearing intracranial U87 glioma cells after various treatment. Anti-CD31 antibody was used to stain neovasculature (C). Quantification of angiogenesis inhibition in tumors with different formulations in comparison to saline group (D). TUNEL kit was used to label apoptosis cells (E). Quantification of TUNEL positive cells in tumors with different formulations in comparison to saline group (F). Anti-CD133 antibody was used to label tumor stem cells (G). Quantification of tumor stem cell inhibition in tumors with different formulations in comparison to saline group (H). Three random fields were selected under a light microscope. Mean ± SD, n = 3. *p < 0.05, **p < 0.01.

Concurrent combination chemotherapy has been emerging as a promising approach to improve the efficacy of cancer therapy 29-31. Thus, ^D^WVAP Micelle/PTL was used in combination with TMZ and ^D^WVAP Micelle/PTX respectively in the present study. For TMZ dosage regimen, mice were randomly divided into four groups (n = 8) and treated with 100 μL of saline, ^D^WVAP Micelle/PTL (5 mg/kg), TMZ (10 mg/kg) and ^D^WVAP Micelle/PTL plus TMZ (5 mg/kg and 10 mg/kg, respectively) via oral gavage or tail vein. Surprisingly, the combination of ^D^WVAP Micelle/PTL (5 mg/kg) and TMZ (10 mg/kg) could dramatically prolong the survival time of nude mice bearing intracranial glioma to 59 days. Compared with TMZ, the combinational therapy prolonged the median survival time by 2.10 times (Figure 6A). For PTX dosage regimen, mice were randomly divided into four groups (n = 8) and treated with 100 μL of saline, ^D^WVAP Micelle/PTL (5 mg/kg), ^D^WVAP Micelle/PTX (6 mg/kg) and ^D^WVAP Micelle/PTX plus ^D^WVAP Micelle/PTL (6 mg/kg and 5 mg/kg, respectively) via tail vein. The combination of ^D^WVAP Micelle/PTL (5 mg/kg) and ^D^WVAP Micelle/PTX (6 mg/kg) could dramatically prolong the survival time of nude mice bearing intracranial glioma to 74 days and improve the survival time by 1.25 times compared with ^D^WVAP Micelle/PTX (Figure 6B). After treatment on the 22nd day, one mouse from each group was sacrificed and the brains were excised for histological study. The frozen sectioned slices of brains were stained with anti-CD31 antibody for assessment of angiogenesis and TUNEL kit for detection of apoptotic glioma cells. The slices were also stained with anti-CD133 antibody to quantify glioma stem cells. As shown in Figure 6C-H, the co-administration regimen could not only increase apoptosis rate in tumor region, but also reduce the number of neovasculature and glioma stem cells.

### Safety evaluation of combination therapy

**Figure 7:**
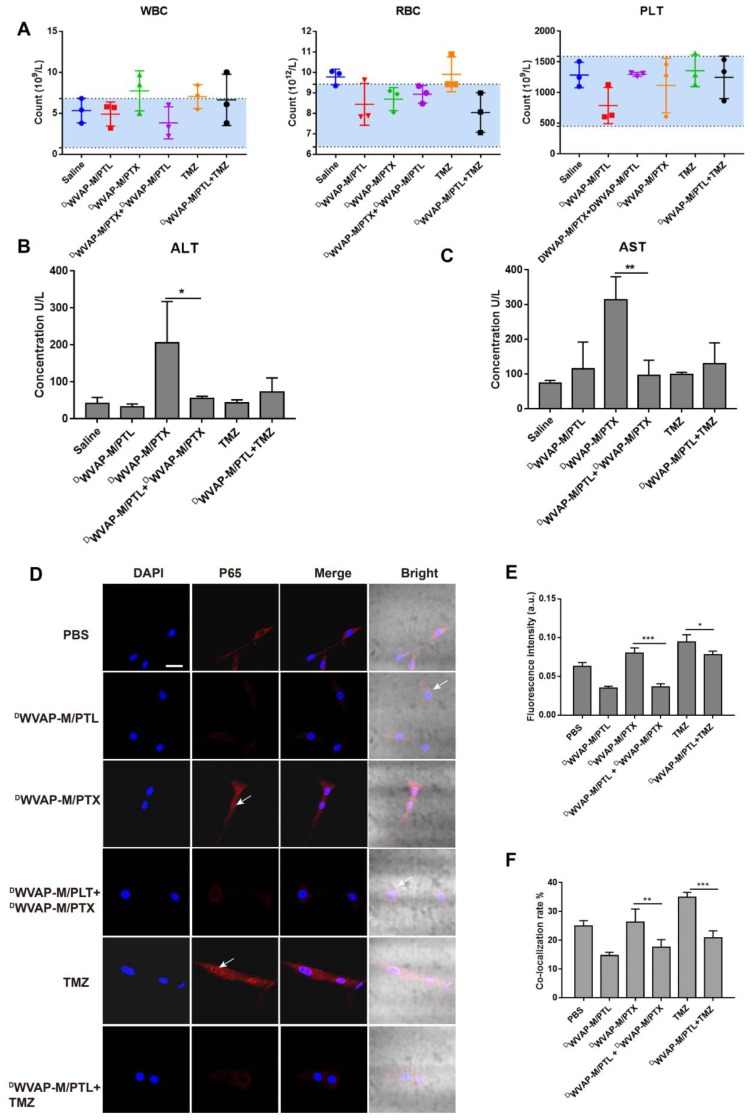
Rationality analysis of combination therapy. Safety evaluation of various treatment regimen. (A) Blood routine parameters of mice with different teatment. Mice (n = 3) were treated with five injections (5 mg/kg for PTL, 10 mg/kg for TMZ and 6 mg/kg for PTX each time) of equal drug dose (at 0, 3, 6, 9 and 12 days). Blue area indicates normal range of each parameter. WBC (white blood cell), RBC (red blood cell), PLT (platelet). Biochemical analysis of blood samples from various formulation treated groups. ALT (Alanine aminotransferase) and AST (Aspartate transaminase) were used to evaluate the liver functions (B and C). Parthenolide used alone or in combination with paclitaxel inhibited the NF-κB activation by inhibiting p65 nuclear translocation. (D) NF-κB subcellular localization was investigated by immunofluorescence staining with anti-p65 antibody (red), the nuclei were stained with Hochest (blue). (E) The fluorescence intensity was used to measure the expression level of p65 by the Image J software. (F) The co-localization rate of blue and red was used to measure the p65 nuclear translocation by the Image Pro Plus software. Mean ± SD, n = 3. * p < 0.05, ** p < 0.01, *** P < 0.001.

Biochemical index examination was performed to investigate whether simultaneous administration of two chemotherapeutic agents could induce extra side effects. Combinational drug therapy did not harm the biochemical functions and ameliorated the liver disfunction induced by PTX (Figure 7B-C and Figure S6). In addition, it would not influence the hematopoietic functions of the mice (Figure 7A), since the WBC, RBC and PLT index of the combinational group were all of no difference with the saline group.

### Parthenolide enhanced the efficacy of Paclitaxel through NF-κB inhibition

Although chemotherapy has benefited numerous glioma patients, more and more reports have showed that recurrence often occurs due to drug resistance. NF-κB plays a pivotal role in tumorigenesis through multiple signaling pathways. Aberrant activation of NF-κB has been observed in various types of cancer. Constitutive activation of NF-κB often causes bad response or resistance to chemotherapeutics, tumor recurrence, and poor outcome of treatment. Conversely, inhibition of NF-κB could sensitize the glioma cells to chemotherapy. The combination of PTL with PTX and TMZ in the present study enhanced anti-glioma efficacy in experimental animals. Since PTL is a potent inhibitor of NF-κB, the inhibitory activities of different PTL formulations on NF-κB pathway were examined. Immunofluorescence was used to detect NF-κB activation and nuclear translocation. After treatment with different formulations for 24 h, cells were processed for confocal fluorescence microscopic imaging. PTL alone or co-administration with paclitaxel or TMZ could cause inhibition of p65 (one of the subunit of NF-κB) nuclear translocation in U87 cells (Figure 7D-F). In addition, we also analyzed the p65 protein level in the nucleus of U87 cells by western blotting (Figure S7) and the results were consistent with immunofluorescence. These results indicated that PTL could potentiate the antitumor activity of PTX and TMZ.

We have previously validated that the versatile peptide (VAP) can achieve active drug delivery to glioma by specifically binding to GRP78 protein 19. However, VAP peptide cannot bypass the blood-brain barrier, which hinders its application in the treatment of the early-stage glioma and its infiltrative region. Therefore, in this study we grafted a brain targeting sequence to VAP peptide to make a “Y-shaped” ^D^WVAP peptide. This strategy is aiming at achieving all-stage precisional glioma targeting. Besides, ^D^WVAP modified micelles do not have immunogenicity and will not influence the drug efficacy as other peptide ligand does such as ^D^CDX and cyclic RGD peptide, making it an ideal ligand for whole process glioma targeting. Paclitaxel loaded nanomedicine could improve chemotherapy for malignant gliomas 32, 33. However, many gliomas are rapidly resistant to the single agent therapies, thus more effective therapeutic options are clinically desirable. Recent studies demonstrated that glioma specimens and cell lines have constitutively high levels of nuclear factor κB (NF-κB) activity 34. Sesquiterpene lactone parthenolide is a potent NF-κB inhibitor, demonstrating satisfactory therapeutic efficacy against glioma with high specificity against glioma stem cells 35. In this study we developed ^D^WVAP functionalized micelles that encapsulated PTL and used it together with TMZ and ^D^WVAP-M/PTX respectively. This two combination regimens not only obtained expected anti-tumor efficacy but also ameliorated the toxicity of chemotherapy, which paved a new way for all-stage precisional glioma targeting.

## Conclusions

In summary, we developed a novel “Y-shaped” all-D peptide (^D^WVAP) that could achieve all-stage precisional glioma targeting. The barrier crossing ability and glioma cell (as well as GSCs) targeting effect of ^D^WVAP peptide were thoroughly investigated and validated. ^D^WVAP peptide modified PEG-PLA micelles were used as nanocarriers to load paclitaxel or anti-GSC agent parthenolide, which were further applied in combination therapy to dramatically boost the anti-glioma efficacy. Our present results highlight the potential of all-stage precisional targeting drug delivery strategy for the treatment of glioma, and the development of ^D^WVAP may pave a new way for targeted glioma therapy in clinic.

## Materials and Methods

### Materials

Boc-protected α-amino acids,^ D^VAP peptide (^D^S^D^N^D^T^D^R^D^V^D^A^D^P^D^C) and ^D^WSW peptide (^D^W^D^S^D^W^D^G^D^P^D^Y^D^S) were purchased from Karebay Biochem co.,Ltd (Ning Bo, China). Methanol, acetonitrile, and other HPLC grade reagents were obtained from Merck. (Darmstadt, Germany). All chemicals were analytic reagent grades. GRP78 protein was supplied by Abcam (Cambridge UK). Biacore series S sensor chips CM5 and HBS-EP buffer were purchased from GE Healthcare BioSciences AB (Uppsala, Sweden). Anti-GRP78 antibody was supplied by ABGENT (Suzhou, China). MPEG_2000_-PLA_2000_ and Mal-PEG_3000_-PLA_2000_ were obtained from Advanced Polymer Materials Inc. (Montreal, Canada). Fluorescein-5-maleimide was purchased from Fanbo Biochemicals (Beijing, China). 5-carboxyfluorescein (FAM) was obtained from Sigma (St. Louis, MO). Rabbit anti-CD31 antibody was purchased from Abcam (Cambridge, MA). Sulfo-Cyanine7 was purchased from LiTTLE-PA Sciences Inc (Wuhan, China). Cy7 (Cyanine 7 maleimide) was purchased from Shanghai Seebio Biotech. (Shanghai, China). Growth factor-reduced Matrigel matrix was supplied by BD Biosciences (San Diego, CA, USA). Palitaxel, temozolomide and parthenolide were purchased from Dalian Meilun Biotechnology Co., Ltd. Rat tail collagen Type I was obtained from Shengyou Biological Technology Co. (Hangzhou, China). DNase I and collagenase were from Dingguo Biological Technology Co. (Shanghai, China). EBM-2 was from Lonza (Visp, Switzerland). DAPI, Apoptosis-Hoechst staining kit and Nuclear Translocation Assay Kit were from beyotime (Jiangsu, China).

U87 cells and human umbilical vascular endothelial cells (HUVECs) were obtained from Shanghai Institute of Cell Biology and were maintained in Dulbecco's Modified Eagle Medium (Gibco) supplemented with 10% FBS (Gibco), 100 U/mL penicillin, and 100 μg/mL streptomycin at 37 °C under a humidified atmosphere containing 5% CO_2_. BCEC cells (primary rat brain capillary endothelial cells) were purified from our previously report 19. OPC cells (oligodendrocyte precursor cells) were kindly provided by Professor Chong Liu in Zhejiang University.

ICR mice, Sprague-Dawley (SD) rats and male BALB/c nude mice of 4-6 weeks age were purchased from the BK Lab Animal Ltd. (Shanghai, China) and raised under SPF conditions. All animal experiments were carried out in accordance with guidelines evaluated and approved by the ethics committee of Fudan University.

### Preparation and characterization of peptides

#### Syntheses and purification of peptides

The “Y-shaped”^ D^WVAP peptide (^D^S^D^N^D^T^D^R^D^V^D^A^D^P^D^C-Ahx-^D^W^D^S^D^W^D^G^D^P^D^Y^D^S) was synthesized via solid phase peptide synthesis method as reported before 36. All three peptides were labeled with Fluorescein dye (Fluorescein-5-Maleimide and Cyanine7-Maleimide) via sulfhydryl-maleimide covalently conjugation. Reaction processes were monitored by HPLC and molecular masses were ascertained by electrospray ionization mass spectrometry (ESI-MS) 19.

#### Binding affinity of ^D^WVAP peptide with GRP78 protein

To investigate whether ^D^WVAP peptide still has binding ability with GRP78 protein, binding analysis was conducted using Biacore T200 system (GE Healthcare). Recombinant human GRP78 protein was coupled to the CM5 sensor chip according to the standard amine coupling procedure. ^D^WVAP, ^D^VAP and ^D^WSW were dissolved in HBS-EP buffer at defined concentrations and injected for recording resonance changes to assess the binding affinity. Data were analyzed using the Biacore software (GE Healthcare) 37.

#### Celluar internalizaiton Assay

Brain capillary endothelial cells (BCEC), U87 cells and HUVEC cells were used to investigate the peptides targeting ability since they were main component of BBB, tumor and tumor vessels respectively 17. OPC cells were used to evaluate the glioma stem cell targeting ability *in vitro* 38. Cellular uptake behavior was observed by the confocal microscope and the fluorescent intensity was captured by flow cytometry 4 hours after pre-incubation with different fluorescein labelled peptides.

#### *In vivo* tumor homing assay

For investigation of peptide tumor targeting ability, the intracranial U87 tumor xenograft orthotropic glioma model (14 days after transplantation) was established as previously reported 22,35. Twelve nude mice were divided into 4 groups, and injected respectively with 200 μL ^D^WVAP-Cy7, ^D^WSW-Cy7, ^D^VAP-Cy7 and free Cy7 in normal saline solutions at a concentration of 0.01 μM through tail vein (n = 3). At predetermined time points, the brain fluorescence imaging was conducted using an *in vivo* imaging system (IVIS Spectrum, Caliper, USA). The fluorescence intensities were quantitatively and qualitatively analyzed via Living Image Software to investigate the distribution of Cy7 labelled peptides.

### Preparation and characterization of functional materials and micelles

Peptide modified functional materials were also synthesized through the covalently conjugation of peptide with Mal-PEG_3000_-PLA_2000_ by a sulfhydryl-maleimide coupling method and excess peptide was removed by dialysis. Micelles were prepared by thin-film hydration method and characterized by dynamic light scattering detector. Meanwhile, the size distribution was confirmed by TEM. Encapsulation efficiency and drug loading capacity were calculated according to previous report 19.

### Tumor homing ability of functional micelles

#### *In vitro* barrier crossing ability

The BBB model was established according to the literature 19,39. The successful establishment was confirmed by measuring the transendothelial electrical resistance (TEER) by an epithelial volt-Ωm (Millicell-RES, Millipore, USA). Monolayers with TEER over 300 Ω•cm^2^ were used for further experiments. For the BBTB model, briefly, HUVECs were seeded in the inserts of transwell and U87 cells were seeded into the lower chamber at the 2:1 HUVECs : U87 ratio and used after three days. Tumor spheroids were established according to previous report 39. Ten days later, the tumor spheroids were transferred to the lower chamber of transwell based BBB model or BBTB model. In each apical chamber, the culture medium was replaced by 50 ng Coumarin 6-loaded micelles of different formulations in DMEM with 10% FBS and incubated for 30 min. Then the tumor spheroids were gently rinsed with warmed PBS for three times, fixed by 4% paraformaldehyde for 30 min and observed by confocal laser microscopy.

#### *In vivo* brain and glioma targeting ability

Biodistribution study was performed in both normal mice and glioma-bearing mice to investigate the biodistribution difference in physiological and pathological conditions. Near-infrared dye DiD was used as a probe and loaded into PEG-PLA micelles for tracking. Healthy mice and nude mice bearing intracranial U87 tumors (14 days after transplantation) were intravenously administered with M/DiD, ^D^WVAP-M/DiD, ^D^WSW-M/DiD and ^D^VAP-M/DiD. Blood was collected and brains were harvested and homogenized in 1 mL PBS at each time point (2 and 24 h after injection, n = 3). The fluorescence intensity of each sample was determined by a Tecan Infinite M200 plate reader. To further observe the distribution of micelles in tumor region, nude mice bearing intracranial U87 tumor (14 days after transplantation) were intravenously administered with M/C6, ^D^WVAP-M/C6, ^D^WSW-M/C6 and ^D^VAP-M/C6. Four hours later, the brains were harvested. After dehydration, brains were frozen in TissueTek® O.C.T. compound. Frozen sections of 10 μm thickness were prepared and stained with 300 nM DAPI for 10 min at room temperature. For analysis, the sections were stained with anti-mouse CD31, rabbit anti-GRP78 antibodies and anti-mouse CD133 followed by Alexa 594 conjugated secondary antibodies to visualize the tumor blood vessels, GRP78 and stem cell respectively. The sections were examined under the confocal laser scanning microscope.

### Anti-glioma efficacy of functional micelles

#### *In vitro* anti-glioma efficacy

MTT assay and destruction of tube formation were performed to evaluate* in vitro* anti-glioma efficacy. For MTT assay, U87 and HUVEC cells were seeded into 96-well plates at a density of 3000 cells per well and cultured overnight. After 24 h cultivation at 37 °C, the cells were treated with different concentrations of drug loaded micelles or free drug alone and then the plates were incubated at 37 °C in a 5% CO_2_ atmosphere. After 72 hours, 20 μL MTT solution was added to each well and incubated for another 4 h. The percentage of cell viability was determined on the basis of absorbance at 490 nm by a microplate reader (Power Wave XS, Bio-TEK, USA). Untreated cells were used as a control. Endothelial cells are able to spontaneously form a capillary-like network on Matrigel *in vitro*, which is an important feature in the process of angiogenesis. Meanwhile, some capillary vessels originating from U87 cells were also developed and defined as Vasculogenic Mimicry (VM). For destruction of neovasculature and VM formation, HUVECs and U87 cells were cultured in the Matrigel as previously reported and then treated with different drug loaded micelles or free drugs 40. After incubation for 12 h, tube structures were observed under an inverted phase contrast microscope (DMI 4000 B, Leica, Germany). For apoptosis analysis, U87 cells were seeded in 12-well plates for 24 h before drug treatment and incubated with the drug for additional 48 h. Attached cells were trypsinized and collected, while culture medium that may contain detached cells was also collected. Both parts of cells were combined and collected by centrifugation at 1000 rpm/min for 5 min. Annexin V positive cells were quantified using an Annexin V-FITC kit by flow cytometry.

#### NF-κB nuclear translocation assay

NF-κB activation and nuclear translocation assay was performed according to the reagent manufacturer's instructions (Beyotime Biotech, China). Briefly, cells were seeded in four-well disk and then treated with different drug loaded micelles or free drug alone or in combinations for 24 h. After washing and fixing, cells were incubated with a blocking buffer for 1 h to suppress non-specific binding. Next, cells were incubated with the primary NF-κB p65 antibody at 4 °C overnight, followed by incubation with a Cy3-conjugated secondary antibody for 1 h, then stained with DAPI for 5 min before observation with confocal microscope. Western blotting analysis was taken to investigate the p65 expression level in the nucleus of U87 cells. U87 cells were treated with different drug loaded micelles or free drug alone or in combinations for 24 h. Nuclear proteins were extracted according to manufacturer's instructions (Beyotime Biotech, China). The protein concentrations were quantified using BCA Protein Assay Kit (Thermo Fisher Scientific). Equal amounts of proteins were subjected to Western blotting analysis using an NuPAGE electrophoresis system.

#### *In vivo* anti-glioma efficacy

The orthotropic glioma model was established as previously reported. The mice were randomly divided into various groups (n = 9) according to different treatment regimen and were treated with different formulations via tail vein after implantation respectively. Survival time was recorded every day and Kaplan-Meier survival curves were plotted for each group. After drug administration, one mouse from each group was sacrificed and the brain was fixed in 4% paraformaldehyde overnight at 4 °C and embedded in paraffin. Immunohistochemistry (including TUNEL assays, CD133 staining and CD31 staining) on paraffin sections were performed as previously reported and observed under an inverted phase contrast microscope. Three equal-sized fields were randomly chosen. The positive cells were counted by image pro plus 5 software.

### Safety evaluation

#### Cytotoxicity and immunotoxicity of functional micelles

Cytotoxicity of ^D^WVAP-PEG-PLA micelle materials was also investigated by MTT assay. ^D^CDX and cyclic RGD peptide both play an important role in brain targeting and tumor targeting respectively. However, their targeting efficacy can be greatly attenuated because of their immunotoxicity after modified onto liposomes 41,42. Therefore, we evaluated the immunogenicity of ^D^WVAP peptide modified micelles through ELISA analysis. ICR mice were intravenously administered with 100 µL Saline (negative control), c(RGDyK)-liposomes (positive control), ^D^WVAP-M,^ D^WSW-M and ^D^VAP-M respectively on day 0, day 3 and day 6. On day 4, day 8 and day 14, the blood of each mouse was collected, and the serum was subsequently derived by centrifugation at 2000 × g for 5 min. Antibody titers in the blood serum were assessed by an indirect ELISA using plates coated with corresponding antigen following a previously reported protocol 42.

#### Biochemical assay of functional micelles

Healthy ICR mice were used to evaluate safety of various formulations. After drug administration, the blood samples were collected and underwent evaluation of biochemical parameters and blood routine parameters by Servicebio (Wuhan, China).

### Statistical analysis

The data were presented as mean ± SD of at least three independent experiments done in duplicate. Representative data were shown. All the data were entered into the Microsoft Excel 5.0, and Graphpad prism 7 software was used to perform the comparison among different groups conducted by two-way ANOVA analysis where appropriate. Differences were considered statistically significant when p < 0.05.

## Supplementary Material

Supplementary figures and table.

## Abbreviations

GSCs: glioma stem cells
PTL: Parthenolide
PTX: Paclitaxel
TMZ: Temozolomide
IHC: immunohistochemical
GBM: Glioblastoma multiforme
BBB: blood-brain barrier
BBTB: blood-brain tumor barrier
VM: vasculogenic mimicry
BCECs: brain capillary endothelial cells
HUVECs: human umbilical vascular endothelial cells
OPC cells: oligodendrocyte precursor cells
WBC: white blood cell
RBC: red blood cell
PLT: platelet
ALT: Alanine aminotransferase
AST: Aspartate transaminase
TEM: transmission electron microscope
TEER: transendothelial electrical resistance
DMEM: Dulbecco's Modified Eagle Medium
SD: Sprague-Dawley
