# Supplementary material for: All-stage precisional glioma targeted therapy enabled by a well-designed D-peptide
Source: Theranostics. 2020 Mar 4;10(9):4073–87. doi: 10.7150/thno.41382 (PMC7086363; doi:10.7150/thno.41382)
Supplement: Supplementary file 1 — Supplementary figures and table. [file thnov10p4073s1.pdf]

Supporting Information

All-stage precisional glioma targeted therapy enabled by a well-designed D-peptide

Danni Ran [a†], Jianfen Zhou [a†], Zhilan Chai [a], Jinyang Li [a], Cao Xie [a], Jiani Mao [a], Linwei Lu [b], Yanyu Zhang [a], Sunyi Wu [a], Changyou Zhan [a, c], Weiyue Lu [a, b, d, *]

[a] Department of Pharmaceutics, School of Pharmacy, Fudan University, and Key Laboratory of Smart Drug Delivery (Fudan University), Ministry of Education and PLA, Shanghai 201203, & State Key Laboratory of Medical Neurobiology and MOE Frontiers Center for Brain Science, Institutes of Brain Science, Fudan University, Shanghai 200032, China.

[b] Department of Integrative Medicine, Huashan Hospital, and Institutes of Integrative Medicine of Fudan University, Shanghai 200041, China

[c] Department of Pharmacology, School of Basic Medical Sciences, Fudan University, Shanghai 200032, & State Key Laboratory of Molecular Engineering of Polymers, Fudan University, Shanghai 200433, China.

[d] Minhang Branch, Zhongshan Hospital and Institute of Fudan-Minghang Academic Health System, Minghang Hospital, Fudan University, Shanghai 201199, China

* Corresponding author: Tel: +86 21 51980006, Fax: +86 215288 0090

Email: wylu@shmu.edu.cn.

†These authors contributed equally to this work.

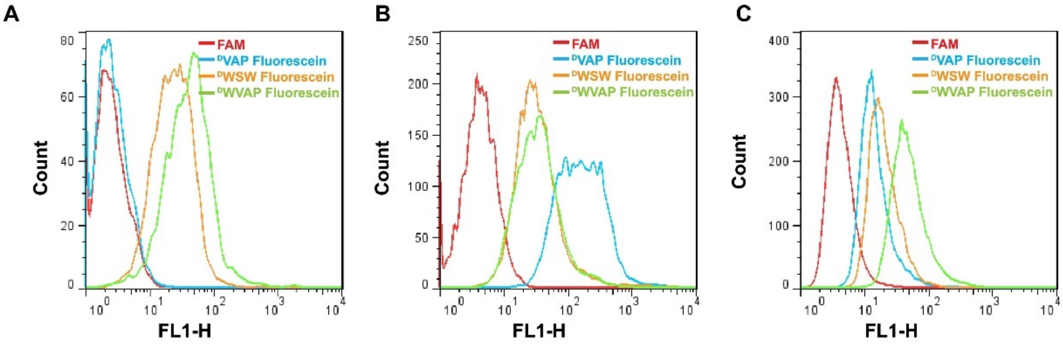

Figure S1. Quantitative cellular uptake was measured by flow cytometry in (A) BCEC cells, (B) U87 cells and (C) HUVEC cells.

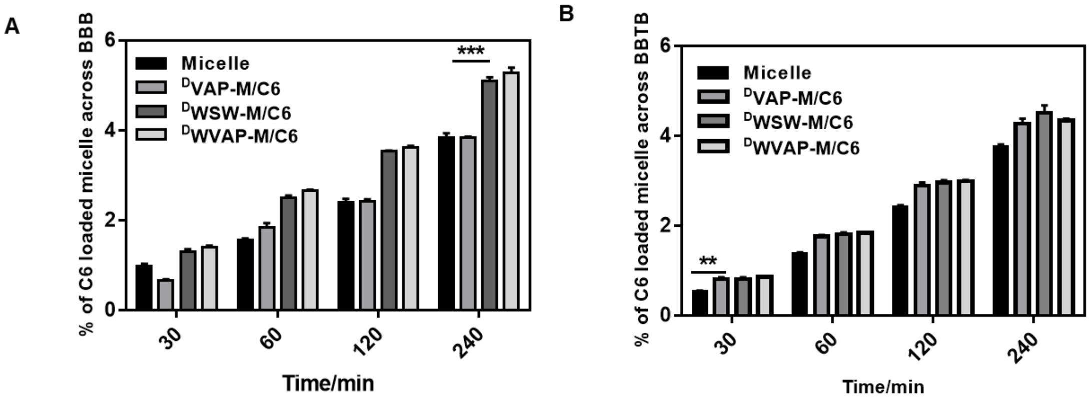

Figure S2. Evaluation of the BBB and BBTB penetrating capacity *in vitro* by using BCECs or HUVEC monolayers. (A) Transcytosis efficiency of Coumarin-6-loaded plain Micelles, ^{D}VAP Micelles, ^{D}WSW Micelles and ^{D}WVAP Micelles on *in vitro* BBB model. (B) Transcytosis efficiency of Coumarin-6-loaded plain Micelles, ^{D}VAP Micelles, ^{D}WSW Micelles and ^{D}WVAP Micelles on *in vitro* BBTB model. Data were presented as mean ± SD, n = 3, ** $p < 0.01$, *** $p < 0.001$.

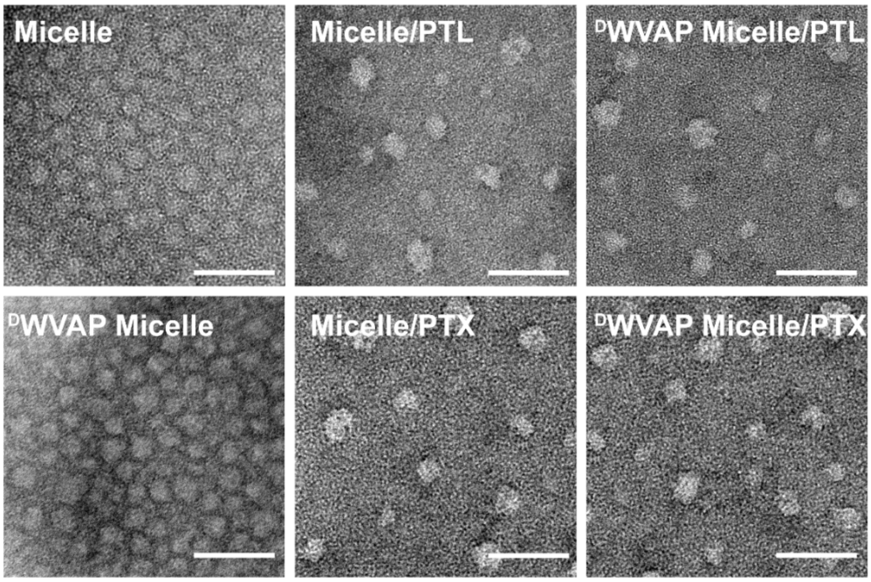

Figure S3. TEM images of ^{D}WVAP peptide modified micelles. (Bar = 50 nm)

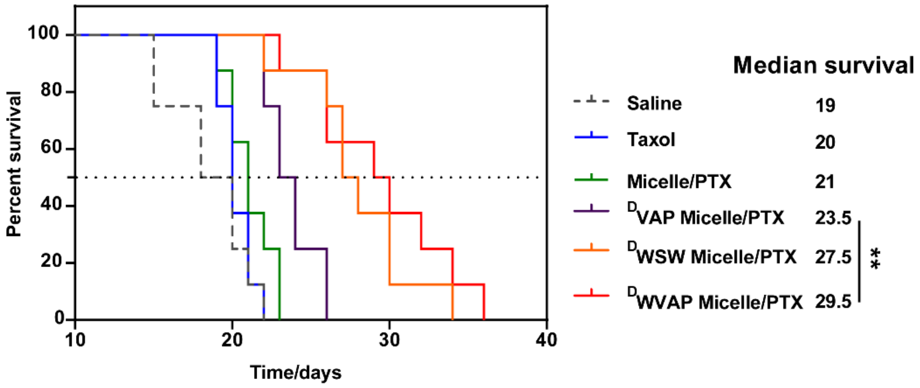

Figure S4. Kaplan–Meier survival curves of intracranial glioblastoma-implanted nude mice treated with different regimens. Mice (n = 8) were treated with four injections (6 mg/kg each time) of equal PTX dose (at 6, 9, 12 and 15 days after glioblastoma implantation). $^{**}p < 0.01$

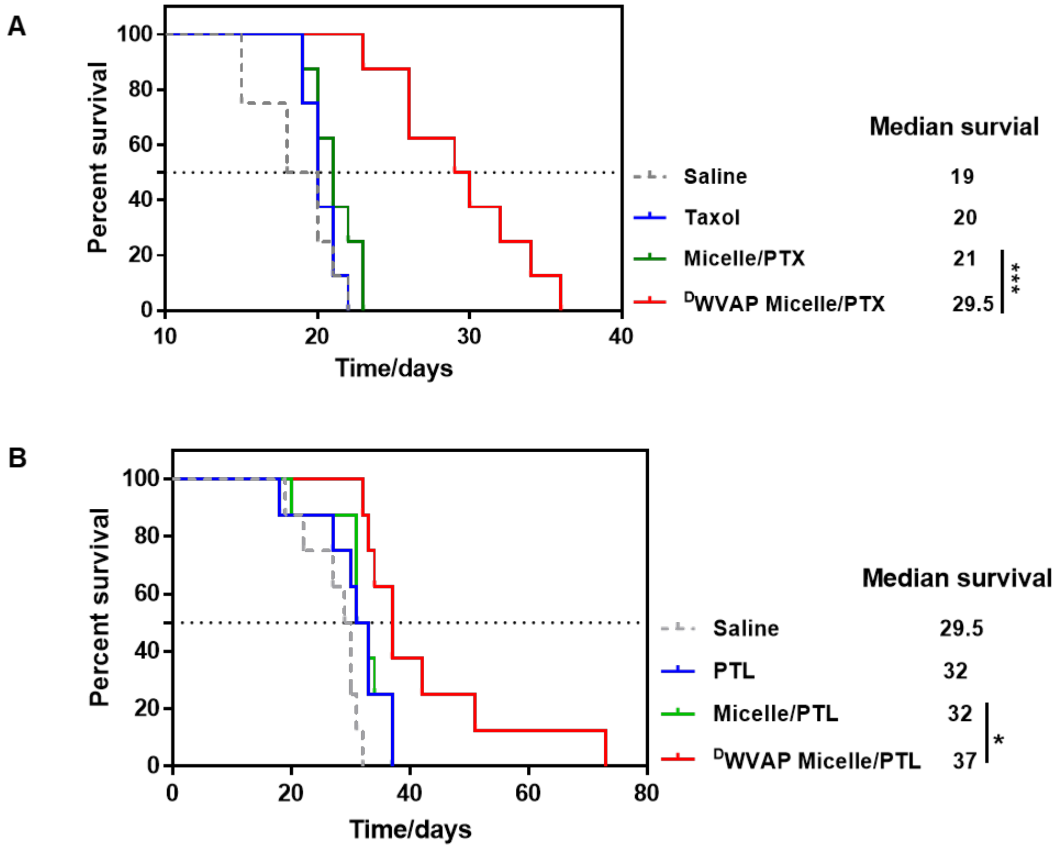

Figure S5. Kaplan–Meier survival curves of intracranial glioblastoma-implanted nude mice treated with different regimens. (A) Mice (n = 8) were treated with four injections (6 mg/kg each time) of equal PTX dose (at 6, 9, 12 and 15 days after glioblastoma implantation). (B) Mice (n = 8) were treated with five injections (5 mg/kg each time) of equal PTL dose (at 6, 8, 10, 12 and 14 days after glioblastoma implantation). *$p < 0.05$, ***$p < 0.001$

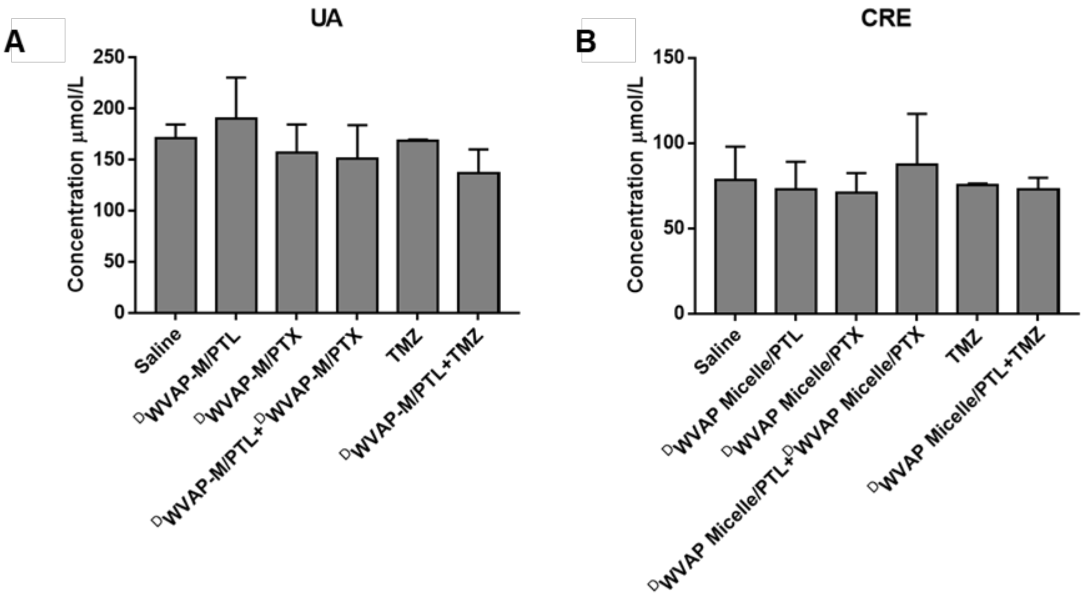

Figure S6. Biochemical analysis of blood samples from various formulation treated groups. UA (Uric acid) and CRE (Creatinine) were used to evaluate the kidney functions (A and B).

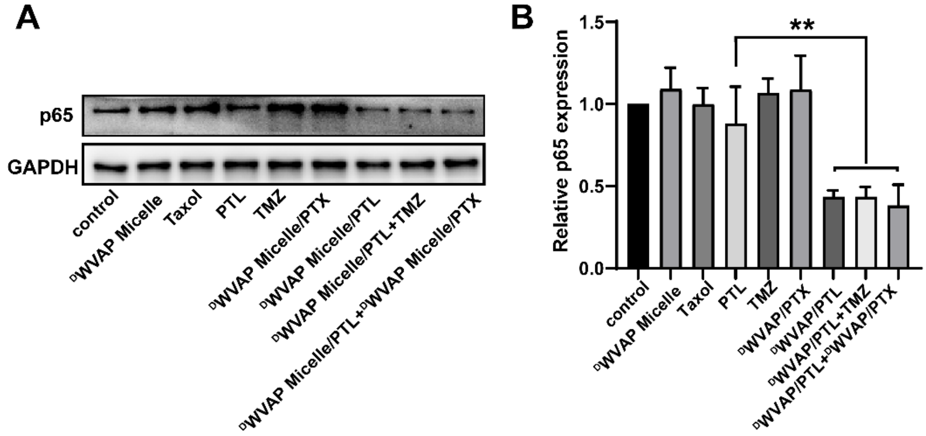

Figure S7. P65 protein expression in the nucleus of U87 cells analyzed by (A) Western blotting, (B) relative protein expression were quantified using ImageJ software. Results were presented as mean ± SD, n= 3, **$p < 0.01$.

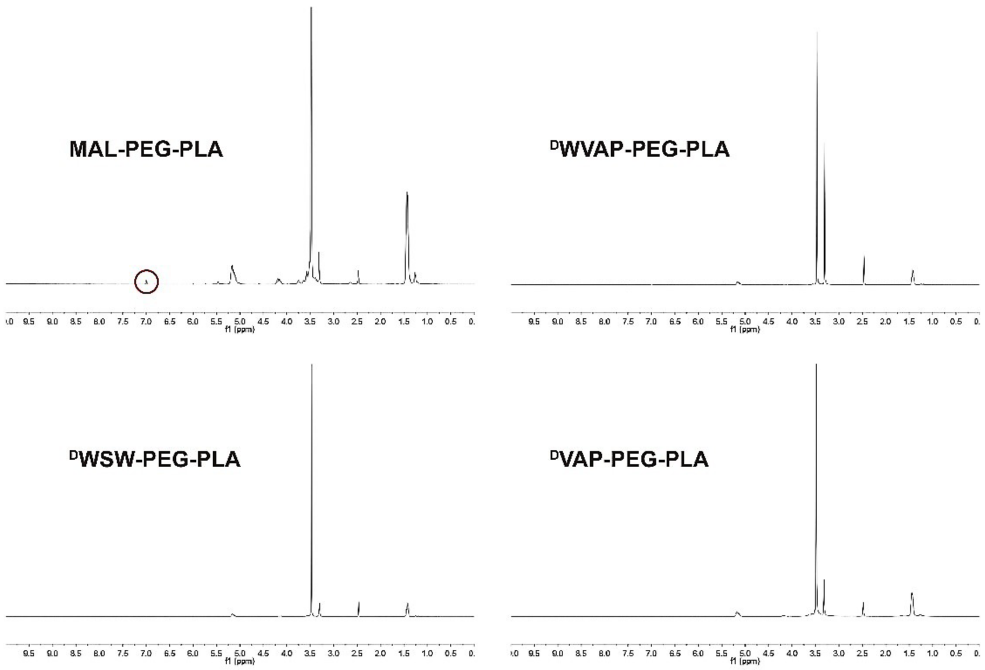

Figure S8. The NMR spectra of Mal-PEG$_{3000}$-PLA$_{2000}$, ^{D}WVAP-PEG$_{3000}$-PLA$_{2000}$, ^{D}VAP-PEG$_{3000}$-PLA$_{2000}$ and ^{D}WSW-PEG$_{3000}$-PLA$_{2000}$. The Mal group presented a sharp peak around 7 ppm in NMR spectrum of Mal-PEG$_{3000}$-PLA$_{2000}$, which disappeared in that of ^{D}WVAP-PEG$_{3000}$-PLA$_{2000}$, ^{D}VAP-PEG$_{3000}$-PLA$_{2000}$ and ^{D}WSW-PEG$_{3000}$-PLA$_{2000}$.

Table S1. Characterization of different micelles (n = 3).

Formulation	Size (nm)	Zeta Potential (mV)	Encapsulation efficiency (%)	Drug loading capacity (%)
Micelle/PTX	29.11 ± 2.59	-2.82 ± 1.10	88.36± 1.84	20.75 ± 0.76
Micelle/PTL	25.69 ± 1.97	-2.86 ± 1.21	87.36 ± 1.62	8.84± 0.51
^{D}WVAP Micelle/PTX	29.25 ± 1.87	-0.53 ± 0.92	84.52 ± 1.82	20.65± 1.29
^{D}WVAP Micelle/PTL	27.60 ± 2.42	-0.55 ± 0.85	83.09 ± 1.80	8.86 ± 0.34
